# Potential therapeutic targets from *Mycobacterium abscessus* (*Mab*): recently reported efforts towards the discovery of novel antibacterial agents to treat *Mab* infections

**DOI:** 10.1039/d1md00359c

**Published:** 2022-03-10

**Authors:** William Addison, Martyn Frederickson, Anthony G. Coyne, Chris Abell

**Affiliations:** Yusuf Hamied Department of Chemistry, University of Cambridge Lensfield Road Cambridge CB2 1EW UK agc40@cam.ac.uk

## Abstract

*Mycobacterium abscessus* (*Mab*) are rapidly growing mycobacteria that cause severe and persistent infections in both skin and lung tissues. Treatment regimens involve the extended usage of complex combinations of drugs, often leading to severe adverse side effects, particularly in immunocompromised patients. Current macrolide therapies are gradually proving to be less effective, largely due to emergence of antibiotic resistance; there is therefore an increasing need for the discovery of new antibacterials that are active against *Mab*. This review highlights recent research centred upon a number of potential therapeutic targets from *Mab* (Ag85C, ClpC1, GyrB, MmpL3 and TrmD), and discusses the various approaches used to discover small molecule inhibitors, in the search for future antibiotics for the treatment of *Mab* infections.

## Abstract 2

Recently reported inhibitors developed against targets from *Mycobacterium absecessus* (*Mab*).

## Graphical abstract

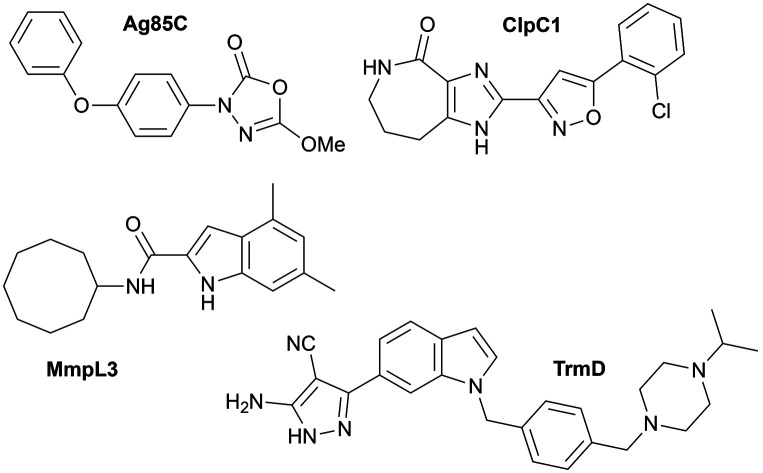

## 1. Introduction and scope

**Fig. 1:**
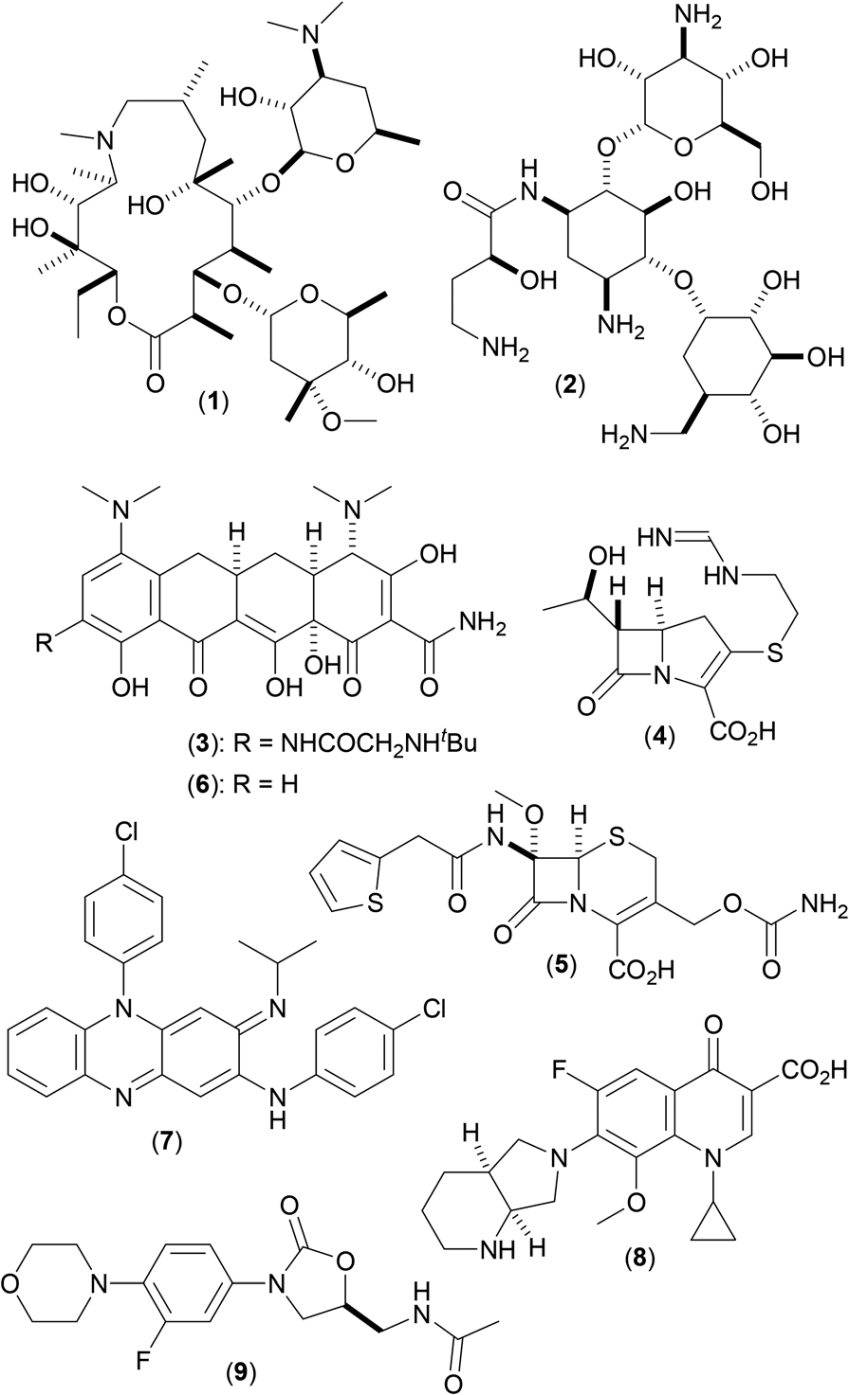
Current drugs used against *Mycobacterium abscessus* infections in cystic fibrosis.

*Mycobacterium abscessus* (*Mab*), first described in 1953,^1^ are a group of non-tuberculous mycobacteria (NTM) named after their ability to produce deep abscesses in human tissues. *Mab* are rapidly growing NTM, that cause severe and slow to repair tissue damage (chiefly in the skin and lungs), and have strong clinical correlation with the progression of decline in lung function in a number of pulmonary disorders, including cystic fibrosis (CF).^2^ Current preferred CF treatment involves prolonged use of a complex cocktail of drugs^3^ including a macrolide antibiotic, such as azithromycin (1), in combination with amikacin (2) and a third antibiotic [one of tigecycline (3), imipenem (4) and cefoxitin (5); or one of minocycline (6), clofazimine (7), moxifloxacin (8) and linezolid (9) (depending on the phase of treatment)] (Fig. 1). Disease progression is often slow,^4^ treatment regimens can take years to be effective, and may result in unwanted side effects, including major organ function impairment (kidney and liver) and sensory disturbances (hearing and sight).^3^

The urgent need for improved treatment opportunities for such difficult to treat conditions has led to an increase in research into the discovery of potential therapeutic targets from *Mab* over recent years, alongside research into the discovery of small molecule inhibitors of these targets as potential new medicines. This review aims to collate recently reported advances within this area of research (within the last 6–8 years of scientific literature), by way of reference to five key protein targets that have been identified (Ag85C, ClpC1, GyrB, MmpL3 and TrmD), and highlights the differing approaches utilized, as well as some of the more important factors that were taken into account, as part of these various studies.

### 1.1. *Mycobacterium abscessus*: form and function

*Mab* are commonly occurring contaminant bacilli of soil and water samples. They are acid-fast, and stain positive using the Gram stain procedure. As with all mycobacteria,^5^ the bacilli are coated with a thick and complex waxy outer membrane that is rich in mycolic acids (and mycolates), rendering them relatively impervious to many antibiotics. *Mab* are rapidly growing mycobacteria (RGM), in stark contrast to other more notorious mycobacteria that have caused major health issues around the globe for centuries; *Mycobacterium leprae* (*Mlep*) and *Mycobacterium tuberculosis* (*Mtb*) [the causative agents of leprosy and tuberculosis (TB) respectively] both have extremely long doubling times. Compared to both *Mlep* (Hansen, 1873) and *Mtb* (Koch, 1882), *Mab* are a more recent discovery^1^ and were only formally recognised as a species^6^ as recently as 1992. Subsequent subdivision of the *Mab* species into three subspecies (*abscessus*, *bolletti* and *massiliense*) occurred as late as 2013, the two major subspecies being *abscessus* and *massiliense*.^7^ Although numerous new *Mab* strains have since been recorded from clinical isolates (yielding additional information regarding whole-genome sequences), in general, knowledge of *Mab* remains relatively scant.

### 1.2. *Mycobacterium abscessus*: mortality and antibiotic resistance

Pulmonary infections caused by a number of closely related non-tuberculous mycobacteria (NTM),^8^ including *Mab*, constitute a serious clinical issue, being associated with high morbidity and mortality.^9^ Limited precise mortality data for *Mab* exists, however a five-year follow-up of the effect of *Mycobacterium avium* (*Mav*) in non-immunocompromised (HIV-negative) patients receiving standardized treatment (in the absence of any macrolide antibiotics), noted a 36% mortality rate.^9^ Prognosis with macrolide therapy is also relatively poor. Five year mortality rates amongst HIV-negative adults, including many who had received macrolides as part of a more complicated cocktail of drugs, have been reported from studies in both Denmark^10^ and Japan^11^ as 40% and 24% respectively.

*Mab* show a high degree of resistance to many antibiotic drugs that might normally be prescribed for the treatment of bacterial infections. The waxy and relatively impermeable *Mab* cell walls act as a natural barrier to the entry of drug molecules into the organism, in a purely physical sense (*via* size exclusion phenomena based upon compound molecular volume) and also physicochemically (due to the highly hydrophobic nature of this heavily lipid laden barrier), so preventing the absorption of drugs that possess lower cLog *P*. Added to this, *Mab* also possess further resistance mechanisms, both intrinsic and inducible,^12^ against xenobiotics that show higher degrees of cell permeability, including several medicinally important macrolides.^13^ A number of varied factors play a role, including target-side modification, efflux and drug inactivation through metabolism.

Target-side modification is a key mechanism responsible for microbial resistance to macrolide therapies, that inhibit bacterial protein synthesis. When bound to their ribosomal target, macrolides prevent the peptidyltransferase from adding the growing peptide attached to tRNA onto the following encoded amino acid and inhibit ribosomal translocation.^14^ Target-side modification, involving either post-transcriptional ribosomal RNA methylation^15^ or gene mutation, results in the macrolide binding with much reduced affinity, thereby decreasing treatment effectiveness against the bacterium.

Efflux of drug molecules constitutes a major issue in the treatment of *Mab* infections. Of the five superfamilies of multidrug efflux pumps that are present in bacteria [ATP-binding cassette (ABC), major facilitator superfamily (MFS), small multidrug resistance (SMR), resistance-nodulation-cell division (RND) and multidrug and toxic compound extrusion (MATE)],^16,17^ categorized based upon the mode of transport and energy coupling methods that they utilize, many are expressed in *Mab* (members of the ABC, MFS and RND superfamilies have been noted),^18^ contributing towards resistance to macrolides^19^ and other antibiotics. In comparison to other mycobacteria, the number of genes in *Mab* encoding for these proteins is high. As a representative example, the mycobacterial membrane protein large (MmpL) family of proteins^20^ are members of the RND superfamily of efflux pumps. Five such proteins have been noted in *Mlep* and fourteen in *Mtb* (H37Rv strain), whereas in *Mab* there are thirty one^21^ (MmpL3, which has been shown to be essential for mycobacterial viability in *Mtb*,^22^ is described below in section 2.4).

Drug inactivation by way of metabolism may occur *via* a number of mechanisms. Drug-modifying enzymes such as β-lactamases, esterases and hydrolases are common, as are other proteins that are able to reduce the effectiveness of xenobiotics by way of processes involving methylation, phosphorylation and specific oxygenation. In mycobacteria, some or all of these mechanisms may be prevalent for a given antibiotic, meaning that some classes of drugs are more at risk that others. The front line drugs are most at risk, and multidrug resistant strains of mycobacteria are becoming more and more prevalent with in the clinical setting. In *Mab*, a number of such processes are known, and have been recently highlighted.^23^

There is therefore an increasingly pressing need for the discovery and development of new antibiotics against a number of key bacterial pathogens. In the case of *Mab*, the highly complex cell structure, coupled with the various important resistance mechanisms outlined above, make the task of discovering new drugs a difficult and complex one. New antibiotics, when they do arise, may sadly have a much shorter timescale of clinical utility that initially expected, due to overuse, with the resulting excessive circulation within the environment leading to acquired resistance. This is a serious problem, and may be even more acute should the antibiotic have only a singular molecular target against which it acts, as only a slight mutation of the target gene or single site modification in the target may be needed to render it effectively useless.

### 1.3. *Mycobacterium abscessus*: general approaches towards the discovery of new therapeutic agents

A number of differing approaches have been used in attempts to discover new therapeutic agents for use in combatting *Mab* infections, generating a variety of lead chemical structures against a wide variety of molecular targets. These approaches can essentially be classified as being of one of two types: those involving initial screening and subsequent drug discovery against a known molecular target (target-based screening approaches), and those where compounds are initially screened against whole-cell mycobacteria and the exact molecular target of the resulting active hit compounds is subsequently identified (phenotypic screening approaches). Both approaches have been successful, and each has a particular set of problems to be overcome to enable compound progression.

Target-based screening approaches are most common, and have proven to be particularly useful in cases where a new target has been discovered using molecular biology and structural biology techniques, when precise structural information regarding the target active site may exist. This is particularly true when the target has been found to be essential for bacterial viability; compounds that inhibit such a target are likely to prove effective when suitably developed into drugs. Such approaches are amenable to a variety of screening processes, either by screening more advanced chemical compounds (from screening libraries or corporate collections), or by using a screening library of much smaller compounds (fragment screening), a powerful technique when used in combination with protein X-ray crystallography, allowing for precise binding information to be used to aid subsequent compound progression. The downside to these approaches is that the inhibitory information gathered may be somewhat artefactual, as it is collected against the isolated target. Affinities of advanced compounds from such approaches are often much reduced when measured against whole-cell bacteria, (due to issues such as a lack of cell permeability, excessive efflux and metabolism of the compounds as described above in section 1.2), compounds having invariably been optimised specifically for binding affinity against the target, with less regard having been placed on them having the correct physiochemical properties for optimal cellular penetration.

In contrast, phenotypic screening approaches involves the screening of libraries of compounds against whole-cell bacteria to ascertain which are able to retard the growth and multiplication of the organism. Compounds that show reasonable potency (low MIC values) may then be further developed in a medicinal chemistry approach that involves progression of the compound to further improve the whole-cell affinity down to the appropriate level. In addition, these approaches require subsequent efforts to allow the identification of the precise molecular target (or targets) against which the compound acts. Possible approaches that allow for effective target validation in mycobacteria include the combination of RNA sequencing, chemoproteomics, morphological profiling and metabolomics.^24^ In most cases, the compounds screened in such approaches are larger and already more developed that in target driven methods (especially when compared to fragment screening), so as to ensure that the cellular potency observed is of the appropriate level. In many cases, the compounds screened in the search for inhibitors of *Mab* by such means are compounds that are already known to be effective against *Mtb*, because of the considerable sequence similarities between all mycobacterial species.^25^ The screening of *Mtb* active compounds tend to give relatively high hit rates,^26^ allowing for the relatively quick and easy identification of active inhibitors against other mycobacterial species, including *Mab*.

## 2. *Mab*: potential therapeutic targets

Multiple approaches might be considered in research efforts aimed at inhibition of *Mab*. Protein targets that are also found in other important mycobacteria are thought to be particularly useful as potential therapeutic targets, as in theory it may prove possible to utilize novel antibiotics discovered by such means in multiple clinical applications. Key individual findings from discovery research might thus be translated between mycobacteria, potentially increasing the rate of compound development. The sheer wealth of literature data that exists from research efforts aimed at more notorious and better understood bacilli (such as *Mlep* and *Mtb*) offers a wealth of such opportunities.

In most cases, particular molecular targets that have been actively pursued have focussed upon those that are believed to be involved in a number of key infective processes, such as bacterial host invasion; as well as those targets that have been shown to be essential for bacterial virulence. In addition, those targets whose function is related to the maintenance of the complex cell superstructure of the bacillus, such as those involved in both the synthesis and transport of the key building blocks necessary for cell wall repair have been deemed worthy of investigation. Likewise, those targets that maintain the biochemical integrity of the mycobacterial cell environment, such as key efflux pumps, have also been seen as targets worthy of pursuit.

We emphasise these ideas below, by way of reference to five potential therapeutic targets from *Mab* that have been the source of such discovery efforts within the last 6–8 years, and highlight key findings in this area, with a particular focus on the distinct classes of chemical structures that these efforts have generated.

### 2.1. Antigen 85C (Ag85C)

Antigen 85 (Ag85), a complex of surface proteins found in mycobacteria, is comprised of three isoform subunits [Antigen 85A (Ag85A), Antigen 85B (Ag85B) and Antigen 85C (Ag85C)]. These structures, which are the most abundantly secreted of mycobacterial proteins, are essential in the maintenance of the complex mycobacterial membrane,^27^ catalysing the reactions that produce both mycolylarabinogalactan (mAG) and trehalose dimycolate (TDM) from trehalose monomycolate (TMM). These transesterification processes, producing the essential highly lipophilic components of the mycobacterial cell envelope, thus play a key role in maintaining the cell impermeability, as well as the infectiousness and viability of the bacterium. A very recent study has explored the binding of the Ag85 complex to fibronectin in multidrug-resistant strains of *Mab*, an important step in host invasion,^28^ thus making this interaction a key factor to be potentially exploited in the design of new drugs against *Mab* infections.^28^

Ag85C is the most active of the three isoforms, the primary function of which is the specific mycolylation of TMM to TDM. Although all three isoforms show a high degree of similarity at the active site regions, they are less homologous at a second distal putative carbohydrate binding site. Molecular dynamics calculations have shown that mutations in the alpha helices located at the ligand entry site can dramatically alter the flexibility of Ag85C in comparison to similar mutations in both Ag85A and Ag85B from *Mtb* (particularly so in the case of Ag85A).^29^ The degree of helical rigidity of both α5 and α9 appears to play a crucial role in both substrate specificity and catalytic activity, and may also be an important factor in intercalation of these proteins with the insoluble cell envelope. X-ray crystal structures of the three *Mtb* isoforms show key differences in the residues at the secondary site which binds trehalose.

A series of twenty seven cyclophostin and cyclipostins analogues,^30^ when screened against mycobacteria from a number of sources, with eight derivatives showing good levels of potency both *in vitro* and *in vivo*.^30,31^ Using both biochemical and structural approaches,^31^ the target for these novel antibacterial compounds was shown to be Ag85C. Fluorescent assays showed a significant loss of fluorescent intensity when a TAMRA-FP fluorescent probe (known to bind to serine dependent proteins such as Ag85C) was incubated with Ag85C that had been pretreated with the cyclic phosphonate derived inhibitors, suggestive of protein phosphonation as a mode of action. This was confirmed by X-ray crystallography,^31^ with structural data highlighting the attachment *via* covalent linkage of the inhibitors to an important catalytic serine residue located at the active site, that is key to the acyltransferase activity of the proteins (a specific residue identified previously^32^ using mass spectrometric techniques).

**Fig. 2:**
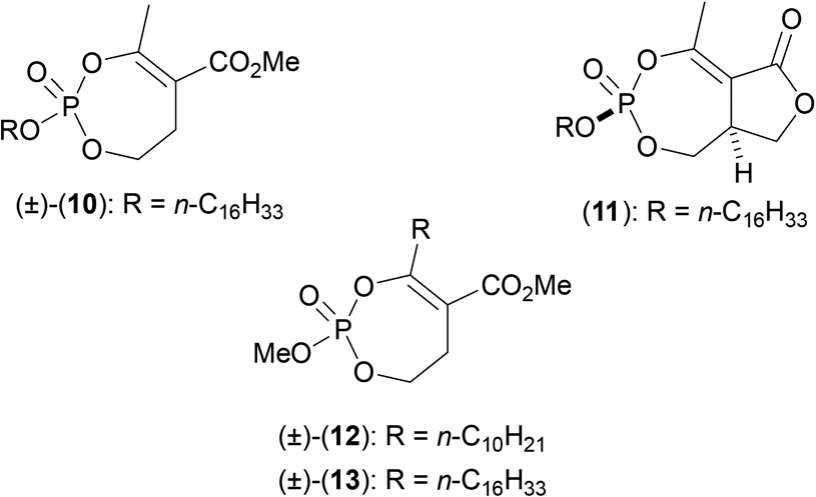
Cyclic phosphate based inhibitors of Ag85C from *Mycobaterium abscessus.*

Subsequently, the series was expanded to thirty eight members, and was rescreened against *Mab*. Four compounds (10–13) were found to be effective to varying degrees (Fig. 2), with (10) proving to be the most active compound (MIC_50_: 370 nM). Activity-based protein profiling (ABPP) experiments in combination with protein mass spectrometry confirmed that these compounds active against serine or cysteine containing proteins in *Mab*,^33^ and were likely acting be affecting lipid metabolism and cell wall biosynthesis. As per previously, experiments involving the use of a TAMRA-FP fluorescent probe confirmed that Ag85C was a major target of these inhibitors in *Mab*, suggesting their mode of action to be similar to that in other mycobacteria previously studied (outlined above). Ag85C has not been confirmed to be the only target in *Mab* against which this important class of compounds acts, and this is promising news, as *Mab* inhibitors that act by way of multiple targets may prove more effective, and may help to reduce the possibility of rapid mutations occurring in the bacillus that allow the organism to acquire resistance to new antibiotics.

**Fig. 3:**
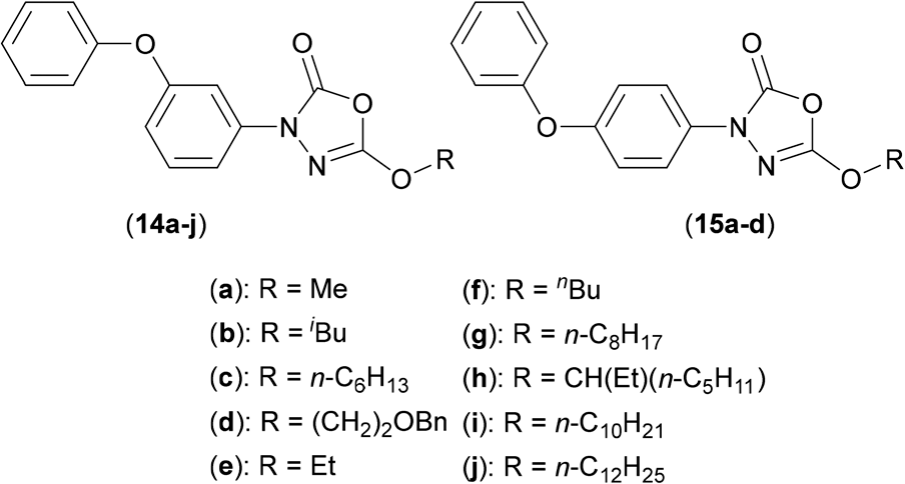
Oxadiazolone based inhibitors of Ag85C from *Mycobaterium abscessus.*

The oxadiazolone class of antibiotic derivatives (14a–j and 15a–d) (Fig. 3), previously found to be active against multiple targets in *Mtb*,^34^ have recently been screened against *Mab*,^35^ to determine whether their possible clinical application might be extended. The lead compound from these studies (15b) (MIC_50_: 33 μM) impaired both intra- and extracellular growth of *Mab*, albeit to only moderate degrees. As outlined above, ABPP experiments were used in combination with mass spectrometric analysis and showed that the activity of the compound was derived from the inhibition of enzymes that utilize either a catalytic serine or cysteine residue. In *Mab*, these enzymes are mainly involved in processes that involve control either lipid metabolism and/or generate the constituent materials required for cell envelope synthesis.

One such target was shown to be Ag85C, with fluorescence based experiments performed with purified *Mab* Ag85C in the presence of (15b) resulting in significantly reduced fluorescence of the TAMRA-FP fluorescent probe used (as detailed above). In addition, significant resistance was observed, resulting in an increased MIC value (by 2–3 fold), upon overexpression of the Ag85C gene.^35^ A possible mechanism of action was elucidated using mass spectrometry studies, the data from which suggested covalent attachment between (15b) and the key catalytic serine residue at the active site of Ag85, so resulting in functional inhibition. Such mechanistic understandings are key to allow the further elaboration of these compounds, in order to target Ag85C more effectively, and so greatly increase antibacterial activity.

### 2.2. Caseinolytic protease chaperone 1 (ClpC1)

Caseinolytic protease proteins are a family of large homologous proteins that are ATP-dependent, first identified in *E. coli* as a two-component ATP-dependent protease (ClpP) comprised of fourteen subunits forming a complex tetradecameric structure from two heptameric rings that form a hollow cylinder, inside of which lie fourteen proteolytic sites. ClpP is highly dependent upon a series of ATPase active chaperone subunits, such as ClpA, ClpX or ClpC, which form key associated complexes.^36^ The ClpP proteins are considered to be valid drug targets, and as ClpC1 is found in Gram-positive bacteria and cyanobacteria, it has been viewed as a promising target in mycobacteria.

**Fig. 4:**
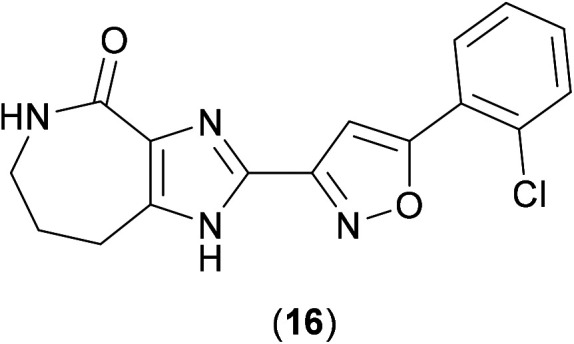
Structure of GSK18 (16), an inhibitor of the ATPase activity of ClpC1.

High Throughput Screening methods (HTS) methods have been used against *Mtb* in order to identify inhibitors of the ClpP1P2 unit. ClpP1P2 is able to degrade small peptides, so this peptidase activity was monitored using a fluorescence-based approach by conjugation of a fluorophore to the hydrolysed peptide and measuring the increase in fluorescence upon hydrolytic release. As these studies aimed to find compounds that bound to the cofactors of ClpP1P2, ATP-dependent assays were also developed. A kinetic assay, in which the degradation of a substrate (GFPssra) was measured by fluorescence, was developed, and validated as a possible HTS option. A significant drop-off in fluorescence would be expected in the presence of ATP.^37^ In order to identify inhibitors that reduce the hydrolysis of ATP by ClpC1, the release of the hydrolysis product (ADP) needed to be measured, using a coupled assay, where ATP is regenerated from ADP using a mixture of pyruvate kinase and lactate dehydrogenase enzymes (which require NADH), the oxidation from NADH to NAD^+^ can be observed by way of a drop in absorbance.^37^ With these suitable assay methods in place, representative members from a library of over 1.8 million compounds were screened *via* these methods (due a limit in the amount of available protein for screening), and from these, GSK18 (16) (Fig. 4) was identified as a novel inhibitor of ATPase activity of ClpC1.

**Fig. 5:**
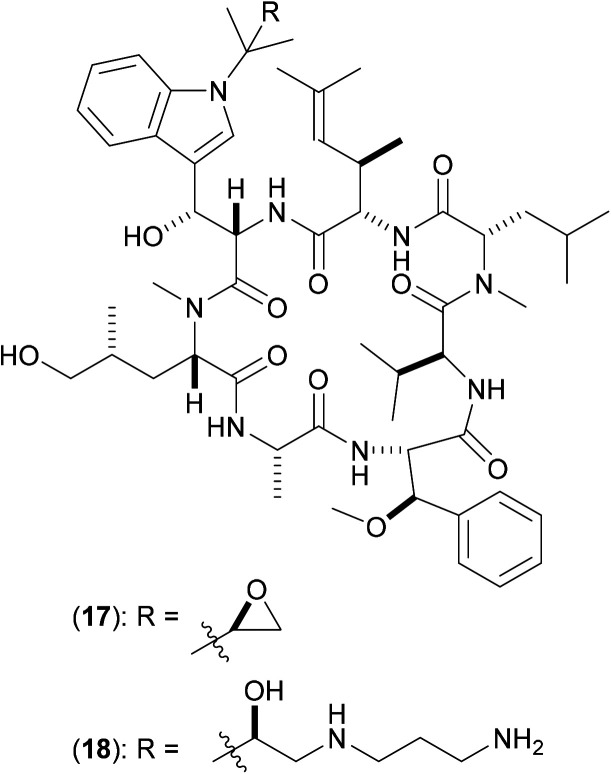
Structures of cyclomarin A (17) and amino derivative (18): inhibitors of ClpC1.

The natural product cyclomarin A (17) (Fig. 5) has been identified as a potent anti-TB agent with some analogues being active against a panel of multidrug-resistant isolates of *Mtb*.^38^ ClpC1 was identified as the target of inhibition *via* affinity chromatography [using the active derivative (18) covalently linked to sepharose beads] in combination with proteomic analysis and isothermal titration calorimetry. ClpC1 is essential for growth in *Mtb*, so extension of these studies against ClpC1 from other mycobacteria is clearly pertinent. Fluorescence studies show that in the presence of cyclomarin A (17) there is an enhanced rate of breakdown of the peptide hydrolysis, indicating that (17) increases the proteolytic activity at the ClpP sites.

The mechanism of action of these analogues against ClpC1 was not determined, but affinity chromatography in the presence of ATP suggests that they do not acting by way of competition with ATP binding;^38^ thus a mode of action involving allosteric binding to the ClpC1 hexameric ring was suggested, since the presence of (17) results in increased proteolysis. A co-crystal structure of (17) bound to the N-terminal domain of ClpC1 has since been published,^39^ and is suggestive of the idea that upon binding, (17) significantly reduces flexibility in this key domain, so resulting in a reduction in the ability of ClpC1 to partially close, and thereby restrict access to, the ClpP protease tunnel, hence allowing greater access of various proteins to the key catalytic machinery contained within, thus resulting in the greater degrees of hydrolysis and degradation that are observed experimentally.

**Fig. 6:**
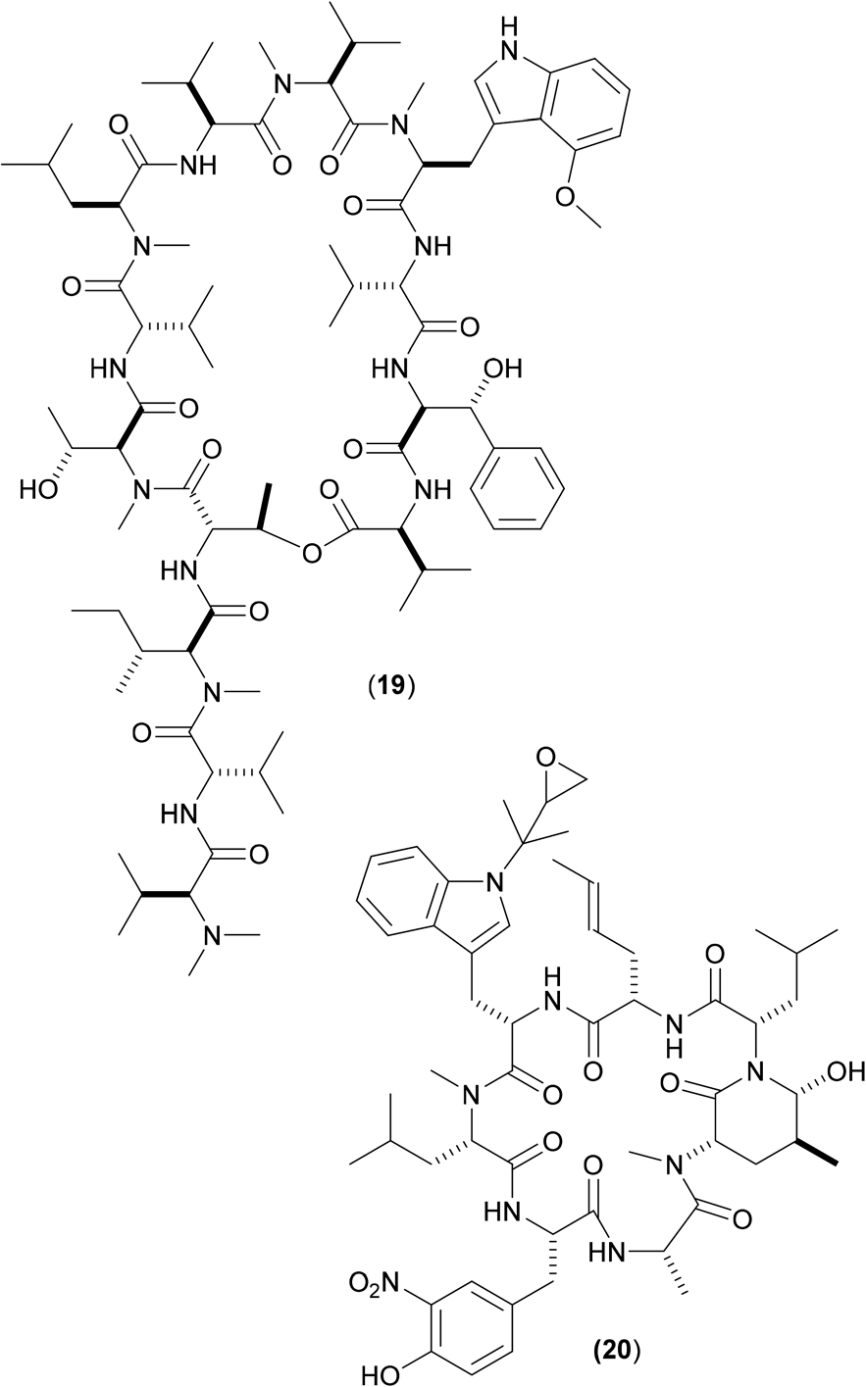
Structures of ecumycin (19) and rufomycin derivative (20): inhibitors of ClpC1.

Other cyclic peptides, such as ecumicin (ECU) (19), are also known to target ClpC1 [(19) MIC against *Mtb*: 160 nM;], as are another class of cyclic peptides, the rufomycins (also known as ilamycins) (Fig. 6). These were initially screened against *Mtb*, with the most potent rufomycin derivative being (20). Subsequently, (17), (19) and (20) were screened against *Mab*. Most notable was that two different mechanisms of action were observed for (19) and (20).^40^ Whereas (19) promoted ATPase activity and uncoupled ATPase activity from proteolytic activity (thereby stopping the enzyme from eliminating proteins), (20) significantly decreased the proteolytic capabilities of the complex, so keeping the ATPase activity relatively constant.

These findings give a further indication as to the promising nature of the target, as they illustrate that there are (at least) two differing opportunities for inhibition. Surface plasmon resonance studies between both (19) and (20) with wild-type full length ClpC1 from *Mab* yielded binding affinity values in the nanomolar region (both approx. 100 nM),^40^ with (20) also shows promising activity (MIC 420 nM) against whole-cell *Mab* (as well as against other key mycobacteria). A co-crystal structure of (20) bound to ClpC1 from *Mtb* has recently been solved;^41^ similar studies involving X-ray crystallographic attempts to determine the precise way in which this pair of compounds interact with ClpC1 from *Mab* might aid understanding as to the divergent inhibitory profile of (19) and (20) noted above, thereby allowing for the design of improved derivatives *via* either inhibitory mechanism.

### 2.3. DNA gyrase B (GyrB)

DNA gyrase is a type II DNA topoisomerase (first isolated from *E. coli* in 1976)^42^ that has an alpha tetrameric structure comprised of two subunits, DNA gyrase A (GyrA) [the molecular target for the fluoroquinolones, such as moxifloxacin (8)] and DNA gyrase B (GyrB), that convert relaxed, closed-circular DNA into negatively supercoiled DNA in order to pack DNA more efficiently in cells. GyrA bears the breakage-reunion active site which is coupled with ATP hydrolysis activity promoted by GyrB. DNA gyrase is the single type II topoisomerase expressed in *Mtb*,^43^ which presumably also carries out similar functions to canonical gyrase and topoisomerase IV. As a result of this apparent multifunctional role, this enzymic system has been touted as a key target in *Mtb*, and by extension, in other mycobacteria, including *Mab*.

**Fig. 7:**
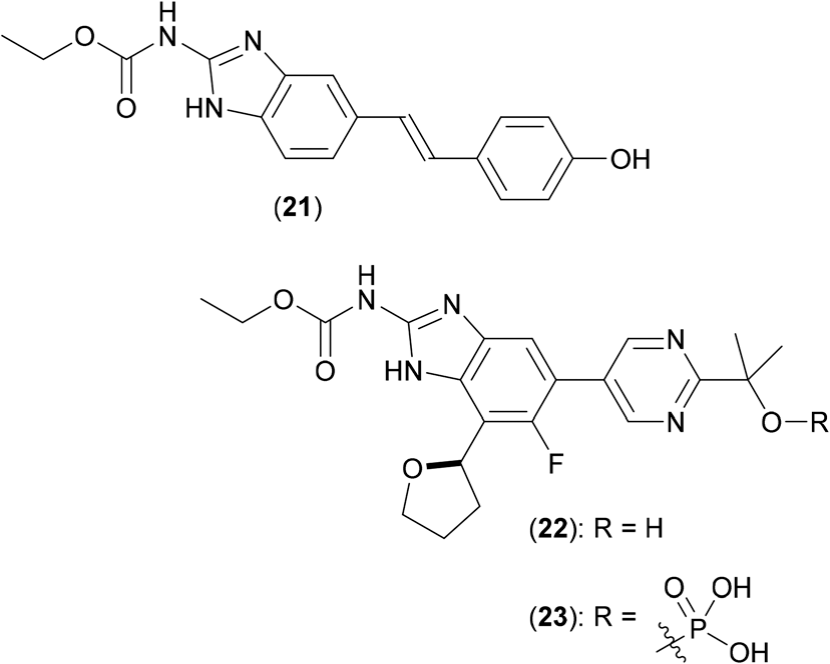
Structures of HTS hit (21), clinical candidate (22) and prodrug (23).

In 2008, a structure-guided approach was used to optimise novel benzimidazole ureas against the ATP binding site of DNA gyrase and topoisomerase IV enzymes in antibiotic resistant strains of key Gram-positive bacteria. The original screening hit, compound (21) (Fig. 7), was discovered by way of a HTS performed using a library of over 30 000 compounds utilizing an ATPase assay.^44^ Extensive structure-guided medicinal chemistry efforts, aimed at improving *in vitro* potency against the enzymes, reducing efflux, and improving cellular penetration led to advanced compounds. Second generation inhibitors followed, with improved molecular profiles, ultimately leading to preclinical candidate (22).^45^

Compound (22) was subsequently screened against a number of mycobacteria,^46^ the dual inhibition of both gyrase and topoisomerase IV activities being seen as beneficial, as a potential aid to help minimise the frequency of resistant mutations (a major problem with current antibiotics). Multiple clinical *Mtb* strains (both drug-sensitive and drug-resistant) were shown to be inhibited by (22), both *in vitro* and *in vivo*, with the phosphate based pro-drug (23) proving to be even more effective that (22). Twenty two clinical isolates of *Mab* were also treated with (22), and growth inhibition noted, yielding MIC values on a par with those shown by both clarithromycin and moxifloxacin (8). Mode of action of (22) in *Mab* isolates was confirmed *via* gene knockout studies, which showed that as expected, showed the effect of the compound to occur by inhibiting the *gyrB* gene.

**Fig. 8:**
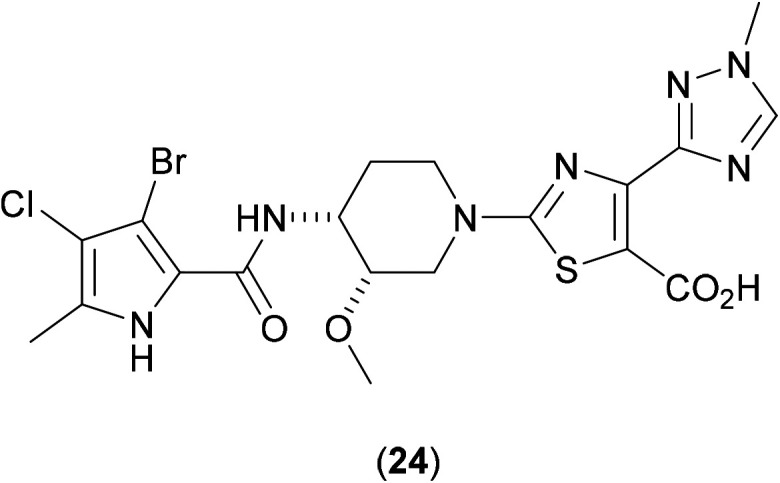
Pyrroloamide (24): a representative of the class of inhibitors of GyrB.

Although no crystallographic information is available of (22) bound to GyrB from *Mab*, possible insight into the precise mode of action of such GyrB ATPase site inhibitors may be gleaned from previous work, in which a series of pyrroloamides^47^ were screened against *Mtb*. By isolating spontaneous resistant mutants and mapping the point mutations produced, a homology model for the GyrB site in *Mtb* was generated, and allowed for the deciphering of the structure–activity relationship (SAR) and binding interactions in mycobacterial GyrB. A co-crystal structure of (24) (Fig. 8) with GyrB (from *M. smegmatis*) showed the compound to bind to the enzyme *via* a water mediated hydrogen bonded interaction with Ser208, a key amino acid identified as the site of the important point mutation (Ser208Ala) that confers resistance.

Despite research efforts against GyrB (and topoisomerase IV) being such a heavily worked area,^48^ little or no clinical success has been achieved. Early work initially centred around infections that showed resistance to penicillins, later other resistant strains, such as methicillin-resistant *Staphylococcus aureus* (MRSA) came to the fore. Only relatively recently have efforts been directed towards mycobacterial related diseases. It is clear that for further progress to be made in this area, particularly against *Mab*, more work still needs to done to aid understanding of the precise binding modes of key compounds that are active against GyrB from *Mab*.

### 2.4. Mycobacterial membrane protein large 3 (MmpL3)

Mycobacterial membrane protein large protein 3 (MmpL3) is a member of the MmpL family of proteins (members of the RND superfamily of efflux pumps of mycobacteria), that are responsible for exporting endogenous lipophilic molecules. Such efflux pumps are heavily expressed in *Mab*, and are a major contributory factor in the poor performance of many antibiotics against mycobacterial infections;^49^ they are therefore seen as important targets for anti-mycobacterial research efforts, particularly so MmpL3, as it essential for the growth and viability of *Mtb*. MmpL3 is large (>100 kDa) and complex, consisting of a periplasmic pore domain and a 12-helix transmembrane domain. It is responsible for exporting the bulky hydrophobic substrates that are essential for the synthesis and maintenance of the cell envelope (involving the translocation of TMM, a precursor to the membrane component TDM, across the plasma membrane);^50^ MmpL3 has also been reported to bind haem.^51^ In addition, inhibitors of MmpL3 have also been shown to act synergistically with a number of key antibiotics,^52^ further strengthening the case of MmpL3 as an important therapeutic target against *Mab* infections.

**Fig. 9:**
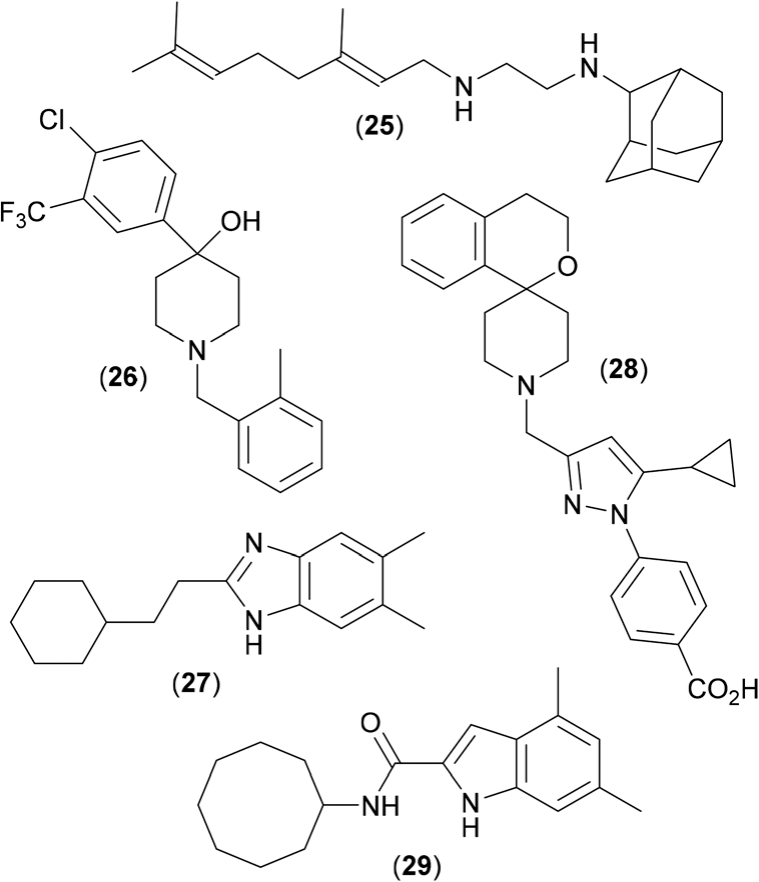
Structures of key inhibitors of MmpL3 from *Mtb* and *Mab.*

A combinatorial solid-phase approach^53^ involving the synthesis and testing of 63 000 closely related analogues of ethambutol with subsequent screening of the most potent compounds against *Mtb*, led to the discovery of a series of adamantyl derived inhibitors, including the antitubercular drug candidate (25)^54^ (Fig. 9), which was shown to be an inhibitor of MmpL3.^55^ A recent phenotypic screening approach translating novel hits against *Mtb* into *Mab* highlighted the piperidinol derivative (26). Whole genome analysis of *Mab* strains resistant to (26) noted several mutations in the gene which encodes for MmpL3, suggesting this to be the target of inhibition.^56^ The study also mapped the mutations which conferred resistance to (26) to generate a 3D homology model with a potential binding pocket.

It is thought that (26) targets specific residues (a pair of Asp and Tyr neighbours) that are essential for the proton relay pathway that provides energy for substrate transport; this key functional tyrosine residue is located in the transmembrane region of the protein and is believed to be conserved in all MmpL proteins.^57^ Likewise, compound (25) is also believed to disrupt proton relay.^58^ Benzimidazole derivative (27) also shows promising anti-mycobacterial activity. Originally discovered as an active in a screen against *Mtb*,^59^ similar activity was recently also noted in *Mab*,^60^ and shown to occur *via* inhibition of MmpL3.^61^ Mapping of resistance mutations (as described above) again suggested that (27) bound to the transmembrane region of the protein, at the neighbouring D/Y amino acid pairs, and so also presumably blocks the proton relay.

A recent X-ray crystal structure^62^ of MmpL3 from *M. smegmatis*, along with co-crystal structures with several inhibitors bound suggests an active site comprised of five distinct regions, four of which are highly lipophilic in nature, with only the central one being polar in nature. This observation undoubtedly explains the binding of structures such as (25–27), all of which have a central protonatable functionality, flanked by highly lipophilic groups. When any of the inhibitors bind, the pair of lipophilic sub-sites in the proton-translocating channel are occupied, further confirming that inhibitors of MmpL3 disrupt the proton motive force for substrate translocation. As the binding region in *M. smegmatis* shares all but four residues with the MmpL3 binding site in *Mtb*, these crystal structures can be seen as useful aids in the rational design of drugs against TB, as well as perhaps in research aimed at combatting *Mab* infections. Other very recent studies^63^ have shown that (28), and other analogous conformationally constrained spiro-derived analogues^64^ are active in *Mtb*, and that very simple indole-2-amides, such as (29), are highly active against *Mab*.^65^ Both (28) and (29) show good activity in the appropriate animal models [(28) MIC against *Mtb*: 660 nM; (29) MIC against *Mab*: 60 nM], and both have been shown to work *via* inhibition of MmpL3,^63,66^ presumably by way of mechanisms akin to those shown by (25–27).

The relative promiscuity of MmpL3 as a target has been commented upon,^66^ having the ability to bind a wide variety of chemical structures, as has the fact that MmpL3 inhibitors are able to work synergistically with several key frontline antibiotics,^52^ (such as rifampicin, bedaquiline, clofazimine and β-lactams), potentially allowing lower doses of these compounds to be administered, so reducing the possibility of the emergence of resistance. MmpL3 is clearly a key target in the area of *Mab* anti-infectives, and X-ray crystal structures of important compounds bound to MmpL3 from *Mab* will no doubt add considerable guiding influence in research in the near future.

### 2.5. tRNA (m^1^G37) methyltransferase (TrmD)

TrmD belongs to the *Mab S*-adenosyl-l-methionine (SAM) dependent methyl transferase family of proteins known as the SpoU-TrmD (SPOUT) RNA methyltransferase superfamily or Class IV methyltransferases.^67^ There are nine other SPOUT enzymes, that recognise different motifs and methylate different positions to TrmD.^68^ TrmD is dimeric, and adopts a trefoil knot motif in order to constrain SAM within the active site.^68^ Its function is to catalyse N1-methylation of tRNA Guanosine 37 (G37) in order to prevent frameshift errors during translation on the ribosome.^70^ For TrmD to function, G36 and G37 must be the substrate RNA sequence on the anticodon loop, so the enzyme can recognise the sequence.^69^ Were these frameshift errors not suppressed by TrmD, protein synthesis would be terminated prematurely, leading to defective cell membrane proteins being produced, leading to cell death.^70^

**Fig. 10:**
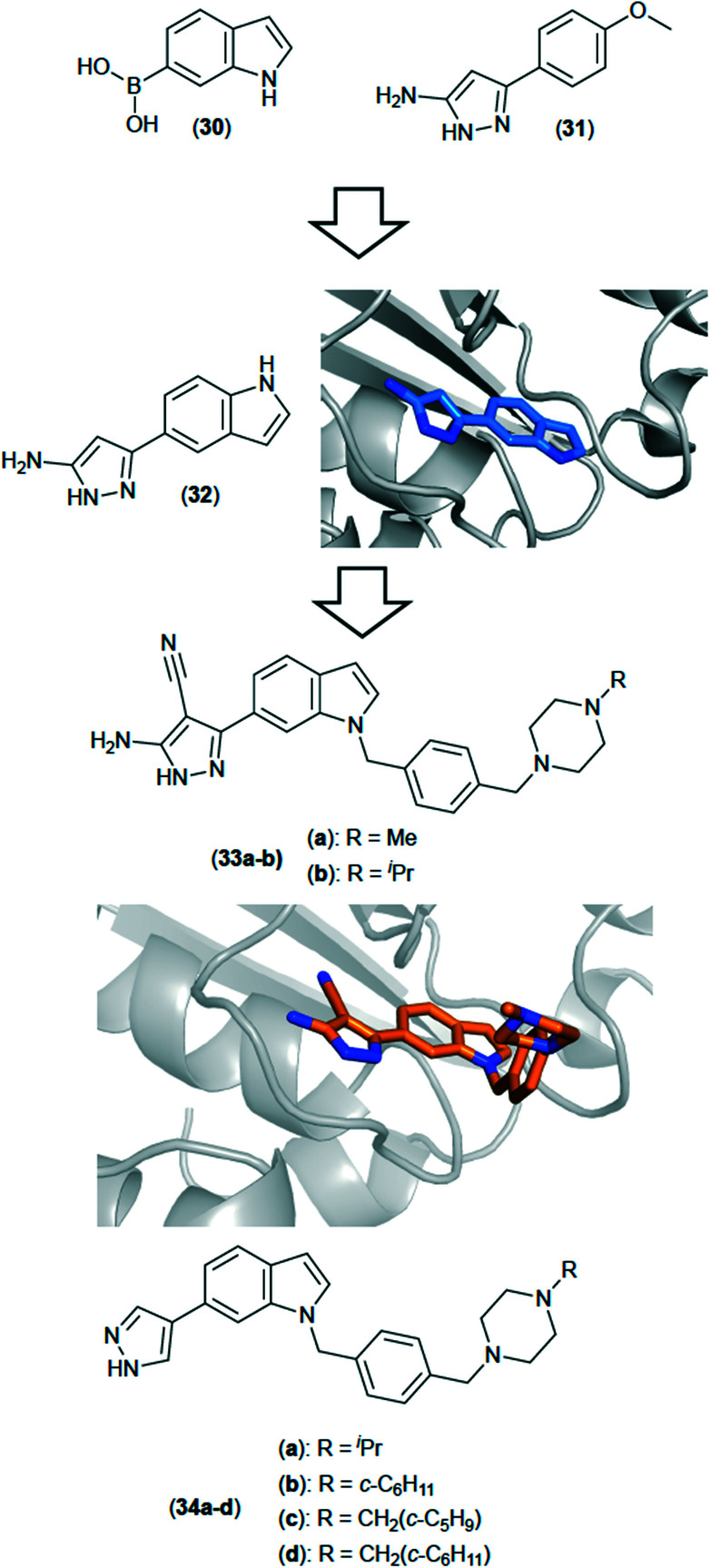
Inhibitors of TrmD from *Mycobacterium abscessus* discovered using FBDD. The initial fragment hits (30) and (32) were merged to give compound (32) (PDB code: 6QQS). Subsequent elaboration led to the development of compounds (33a) and (33b). X-ray crystal structure is of compound 33a (PDB: 6QR8).

A structure driven approach^71,72^ involving a fragment based drug discovery (FBDD) strategy has been utilised against TrmD from *Mab* to discover a series of novel inhibitors (Fig. 10). FBDD is an efficient way of screening, using a relatively small library of compounds to explore a large amount of chemical space. Gene transposition studies showed TrmD to be essential for growth in *Mab*, and X-ray crystallographic studies afforded an apo structure for full-length TrmD from *Mab* as well as structures with SAM bound, thus indicating the substrate binding pocket. A fragment screen using a library of 960 fragments yielded fifty three hits in a differential scanning fluorimetry (DSF) based assay, of which twenty seven showed some electron density in the active site when soaked into apo crystals of TrmD, the key interactions being between the ligand in the area in which the adenine base of SAM binds.^71^

Overlap of multiple X-ray structures of the weakly binding fragment hits gave rise to a clearer understanding of factors that that affected binding. Binding affinities were determined using isothermal titration calorimetry (ITC), in order to rank (and prioritise) fragment hits. A fragment merging strategy involving hits (30) (*K*_d_: 260 μM) and (31) (*K*_d_: 170 μM) was pursued, to generate (32) (*K*_d_: 110 μM) which led to more potent analogues. X-ray crystallography was used heavily throughout, and the combination of multiple X-ray structures with detailed SAR data allowed relatively rapid compound progression, resulting in a series of analogues that included lead compound (33a and 33b), which showed low nanomolar affinity against the target.^72^ The series showed reasonable potency against whole-cell *Mtb*, but much less so against *Mab*, possible due to poorer penetration in *Mab*, or due to much increased levels of efflux. Subsequent studies in which focus was more heavily weighted towards factors affecting the overall compound molecular profile led to improved compounds (34a–d), with a more balanced profile between whole-cell *Mab* and *Mtb* (unpublished results).

TrmD from *Mab* is clearly an attractive target for further exploration due to the wealth of X-ray crystallographic data that has been generated, and the fact that it has been shown that compounds with affinities in the nanomolar range can be discovered and subsequently developed, although perhaps with a greater emphasis needing to be placed on properties more pertinent to later stage compounds.

## 3. Discussion

The targets highlighted above, although not an exhaustive list, offer a general representation of key compounds (and methods) described within the recent literature as showing activity against *Mab*. A recent review has highlighted a more comprehensive efforts to find inhibitors of *Mab*, this included both those from target-guided and phenotypic screening approaches.^73^ The variety in both the chemical structures and the molecular size of the compounds highlighted in this review (Fig. 1–10) is worth noting, and stems very much from the origin of the discovery efforts.

Compounds with a much smaller size tend to originate in target directed campaigns, such as that described above against TrmD. Much larger compounds, such as the cyclic polypeptides mostly derive from attempts aimed at translating known activities from one mycobacterium to another (*e.g.* from *Mtb* to *Mab*), whereas intermediate sized compounds can mostly be traced back to the output from HTS campaigns.

**Fig. 11:**
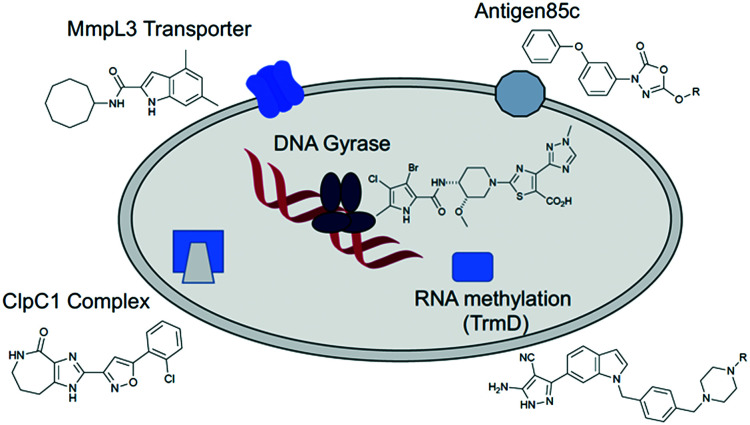
*Mycobacterium abscessus* drug targets discussed in this review and the small molecules acting upon them.

All of these possible approaches are valid, and all have been shown to deliver results in the area, with varying degrees of success. Each approach has inherent strengths and weaknesses associated with it, and none is necessarily superior to the other, although some have generated more interest within the scientific community (and hence more data) than others. It has been noted recently^74^ that all of most promising compounds currently in development against *Mtb* infections are derived from HTS screening efforts involving phenotypic readout from whole-cell assays. Unfortunately, these efforts are not necessarily immediately translatable from bacterium to bacterium; compounds that are highly active against *Mtb* are often far less effective against *Mab*. A recent attempt to analyse the molecular properties of active compounds against *Mtb* has concluded that they invariably all come from a relatively limited area of compound space.^75^ The fact that many would appear to be derived from the same poorly soluble and toxic skeletons may explain the fact that many late stage failures still occur. Undoubtedly compounds with higher lipophilicities than usual are required for *Mtb* and *Mab*, due to the highly lipophilic nature of the bacterial cell wall, cell penetration being an important factor (Fig. 11).

So what constitutes a valid target against *Mab*, and what constitutes a favourable molecular property in an active compound? Clearly those targets that are essential, that are necessary for the growth or invasive property of the mycobacterium must be seen as worth pursuing. In addition, those targets that are located within specific, more readily accessible regions of the bacterium must also be seen as important (for example Ag85C, which is an extracellular target, for which inhibitors do not need to penetrate the complex cell membrane). Those targets for which inhibitors can work synergistically with approved antibiotics (such as MmpL3) may be key to reducing resistance, as lower doses of the clinical antibiotics may be used, resulting in a lack of general environmental exposure of these vital important medicines. Also important are those targets for which compounds might work *via* a number of mechanisms (a prime example being ClpC1), again reducing the likelihood of resistance. Compounds that hit multiple molecular targets might be seen as preferable to those that are target specific; simple mutations may cause resistance to new medicines if the compound is to target specific. Above all, compounds developed need to maintain a high degree of control over physicochemical properties, especially for those that target intracellular proteins.

Phenotypic screening approaches are key, and afford an immediate indication as to the effectiveness (or otherwise) of compounds against whole-cell bacteria, but offer no information related to target engagement. In sharp contrast, with target-based approaches, target engagement is assured, but with no indication as to the ultimate effectiveness of compounds against the bacterium, often resulting in research efforts which prove to be fruitless. Thus phenotypic and target-based approaches might be considered to deliver orthogonal research outputs. In target-based studies, structural characterisation, such as X-ray crystallography, is important, and delivers a wealth of specific information on the binding between ligand and target (such as TrmD), allowing for rapid compound development. It is important, however, that the crystallography is performed using a protein construct that is as close to the natural situation in *Mab* as is possible; surrogate systems, including heavily truncated protein constructs, as well as those from closely related bacteria (very often *Mtb*) are extremely valuable, but are non-ideal, and can only ever deliver an imperfect degree of understanding.

One possible way in which the two seemingly orthogonal approaches of target-based and phenotypic screening might yield improved compounds is to combine the use of both at a very early stage in the overall screening cascade, so utilising high-throughput X-ray crystallographic screening in conjunction with whole-cell assay readout for all compounds that are to be screened. Under such circumstances, X-ray crystal structures would offer assurance of target engagement whilst whole-cell activity greatly reduces the risk of failure of more developed hits at a later stage in the development cascade, with only those screening compounds that deliver high-quality X-ray crystallographic co-crystal structures between protein and ligand and also show acceptable phenotypic activity in a whole-cell assay being chosen for further development, and all other compounds being rejected for further consideration.

## Conclusions

Over recent years, research into the discovery of new antibiotics that are effective against diseases caused by mycobacterial infection have burgeoned, aided by technologies that have allowed whole genome sequencing of individual organisms, allowing for the discovery of new molecular targets and for them to be explored. The continued emergence of multidrug resistant strains within the clinical setting has further increased the urgency of need for therapeutic advances in this area.

Most research over the past few decades has naturally focused upon *Mtb*, the causative agent of TB, that continues to seriously affect millions of individuals worldwide,^76^ more recently *Mab* has come to the fore, due to the increasing prevalence of this bacterium in conditions such as cystic fibrosis, where therapeutic options are very limited and the prognosis for patients is often very poor.

In this review we have attempted to highlight some recent efforts within the field of *Mab* antibiotic research, covering the scientific literature published with the last 6–8 years, concentrating on five protein targets that show promise, and highlighting the successes and pitfalls of the individual approaches that have been taken in each case to further the discovery of potential new medicines. Success rate in the field of antibiotic research remains poor, nevertheless it is hoped that such continued research efforts may eventually bear fruit, and lead to new clinical treatments to aid in the fight against the diseases caused by these difficult to combat mycobacteria.

## Conflicts of interest

There are no conflicts to declare.

## Supplementary Material
